# Supplementary material for: Curated and harmonised transcriptomics datasets of interstitial lung diseases
Source: Data Brief. 2025 Oct 14;63:112139. doi: 10.1016/j.dib.2025.112139 (PMC12581653; doi:10.1016/j.dib.2025.112139)
Supplement: Supplementary file 1 [file mmc1.zip › Supplementary_material/RNA-seq/fastQC reports/GSE180415/SRR15194701_2_fastqc.html]

SRR15194701\_2.fastq.gz FastQC Report 

FastQC Report

Wed 25 Oct 2023  
SRR15194701\_2.fastq.gz

## Summary

- Basic Statistics
- Per base sequence quality
- Per sequence quality scores
- Per base sequence content
- Per sequence GC content
- Per base N content
- Sequence Length Distribution
- Sequence Duplication Levels
- Overrepresented sequences
- Adapter Content

## Basic Statistics

| Measure | Value |
| --- | --- |
| Filename | SRR15194701\_2.fastq.gz |
| File type | Conventional base calls |
| Encoding | Sanger / Illumina 1.9 |
| Total Sequences | 38790849 |
| Sequences flagged as poor quality | 0 |
| Sequence length | 35-80 |
| %GC | 56 |

## Per base sequence quality

## Per sequence quality scores

## Per base sequence content

## Per sequence GC content

## Per base N content

## Sequence Length Distribution

## Sequence Duplication Levels

## Overrepresented sequences

| Sequence | Count | Percentage | Possible Source |
| --- | --- | --- | --- |
| GGGGGGGGGGGGGGGGGGGGGGGGGGGGGGGGGGGGGGGGGGGGGGGGGG | 80299 | 0.20700500780480466 | No Hit |
| GGTGGCGCGTGCCTGTAGTCCCAGCTACTCGGGAGGCTGAGGCTGGAGGA | 76387 | 0.1969201550602824 | No Hit |
| GATCAATCGCCCCCGGGGGTGCCTCCGGGCTCCTCGGGGTGCGCGGCTGG | 73767 | 0.19016598476614935 | No Hit |
| GGTGGCGCGTGCCTGTAGTCCCAGCTACTCGGGAGGCTGAGGTGGGAGGA | 66931 | 0.17254327173916714 | No Hit |
| GCGGTGGCGCGTGCCTGTAGTCCCAGCTACTCGGGAGGCTGAGGCTGGAG | 57454 | 0.1481122519385951 | No Hit |
| GTTCTGGGCTGTAGTGCGCTATGCCGATCGGGTGTCCGCACTAAGTTCGG | 57399 | 0.14797046592097016 | No Hit |
| CCGGGCTCCTCGGGGTGCGCGGCTGGGGGTTCCCTCGCAGGGCCCGCCGG | 54531 | 0.14057696958372837 | No Hit |
| CGGTGGCGCGTGCCTGTAGTCCCAGCTACTCGGGAGGCTGAGGCTGGAGG | 54410 | 0.14026504034495352 | No Hit |
| GCGGTGGCGCGTGCCTGTAGTCCCAGCTACTCGGGAGGCTGAGGTGGGAG | 49121 | 0.12663038130462162 | No Hit |
| CGGTGGCGCGTGCCTGTAGTCCCAGCTACTCGGGAGGCTGAGGTGGGAGG | 48477 | 0.12497019593461334 | No Hit |
| TTTCTATAGTGTGTTACTAGAGAAGTTTCTCTGAACGTGTAGAGCACCGA | 39377 | 0.10151105483667036 | No Hit |

## Adapter Content

Produced by FastQC (version 0.11.7)
